# Multi-Functional OCT Enables Longitudinal Study of Retinal Changes in a VLDLR Knockout Mouse Model

**DOI:** 10.1371/journal.pone.0164419

**Published:** 2016-10-06

**Authors:** Marco Augustin, Stanislava Fialová, Tanja Himmel, Martin Glösmann, Theresia Lengheimer, Danielle J. Harper, Roberto Plasenzotti, Michael Pircher, Christoph K. Hitzenberger, Bernhard Baumann

**Affiliations:** 1 Center for Medical Physics and Biomedical Engineering, Medical University of Vienna, Vienna, Austria; 2 Core Facility for Research and Technology, University of Veterinary Medicine Vienna, Vienna, Austria; 3 Division of Biomedical Research, Medical University of Vienna, Vienna, Austria; Tufts University, UNITED STATES

## Abstract

We present a multi-functional optical coherence tomography (OCT) imaging approach to study retinal changes in the very-low-density-lipoprotein-receptor (VLDLR) knockout mouse model with a threefold contrast. In the retinas of VLDLR knockout mice spontaneous retinal-chorodoidal neovascularizations form, having an appearance similar to choroidal and retinal neovascularizations (CNV and RNV) in neovascular age-related macular degeneration (AMD) or retinal angiomatous proliferation (RAP). For this longitudinal study, the mice were imaged every 4 to 6 weeks starting with an age of 4 weeks and following up to the age of 11 months. Significant retinal changes were identified by the multi-functional imaging approach offering a threefold contrast: reflectivity, polarization sensitivity (PS) and motion contrast based OCT angiography (OCTA). By use of this intrinsic contrast, the long-term development of neovascularizations was studied and associated processes, such as the migration of melanin pigments or retinal-choroidal anastomosis, were assessed in vivo. Furthermore, the in vivo imaging results were validated with histological sections at the endpoint of the experiment. Multi-functional OCT proves as a powerful tool for longitudinal retinal studies in preclinical research of ophthalmic diseases. Intrinsic contrast offered by the functional extensions of OCT might help to describe regulative processes in genetic animal models and potentially deepen the understanding of the pathogenesis of retinal diseases such as wet AMD.

## Introduction

Animal models are widely used in preclinical and basic research of ophthalmology. Particularly mice and rats are beneficial due to their small size and relatively short life cycle, thus accelerating the study of genetic processes and their linked physiological impact [1–5]. Rodent models enable studies of various aspects of ophthalmic diseases such as age-related macular degeneration (AMD) [5–8] or glaucoma [9, 10] and can potentially lead to a better understanding of their underlying pathophysiological processes.

Optical coherence tomography (OCT) is a non-invasive optical imaging method providing high resolution, three dimensional (3D) structural contrast based on the light backscattered from different ocular structures. Since its first publication in 1991 [11], the popularity of OCT for clinical ophthalmic applications has increased steadily. More recently, functional extensions of OCT have been employed to access additional contrast and gain functional information of biological samples. OCT based technology was used in the eye to visualize and quantify blood flow (OCT angiography (OCTA) [12–14], Doppler OCT [15, 16]), to determine optical polarization properties related to micro-structure such as melanin pigments (polarization sensitive (PS) OCT [17, 18]) or to access blood metabolism related parameters such as blood oxygen saturation (spectroscopic OCT [19]). Apart from the eye, functional extensions of OCT were developed to study biomechanical tissue properties using optical coherence elastography [20].

Resolution in the micrometer range, non-invasiveness and the intrinsic contrast provided by the functional extensions make OCT an interesting technology for longitudinal studies. Nevertheless, in preclinical research of small animals OCT only plays a minor role. Custom-made OCT systems tailored for small animal imaging were used in the past to study structural and functional properties in rodent eyes. The retinal thickness in rats and mice was investigated for instance by Hariri et al. [21] and Srinivasan et al. [22]. Morphological changes in retinal vascular plexuses were studied using OCTA [23], while total retinal blood flow was investigated by Zhi et al. [24] and Choi et al. [25]. Quantification of the blood oxygen saturation was recently shown using visible-light OCT by Chen [26] et al. and Soetikno et al. [27].

In our group, PS-OCT was recently translated to rodent imaging to study the optical properties of retinal tissue microstructure such as the birefringence in the nerve fiber layer (NFL) [28] and depolarization caused by melanin pigments in the choroid [29].

**Fig 1:**
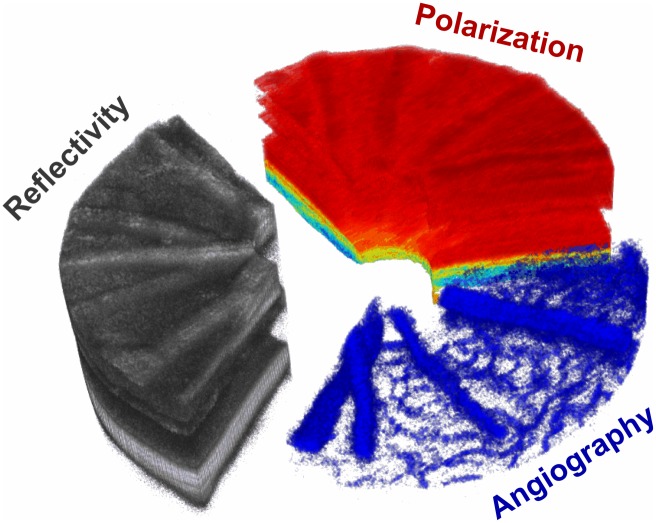
Multi-functional OCT. While OCTA allows the visualization of blood flow non-invasively, PS-OCT gives additional intrinsic contrast of tissue with certain microstructures such as melanin pigments (yellow to blue color). In this work, these two functional extensions are extending the conventional OCT approach to multi-functional OCT for small animal imaging.

In this work, we present a multi-functional OCT approach to image the posterior mouse eye with a threefold contrast comprising reflectivity, PS contrast and OCTA. An exemplary image illustrating this threefold contrast is shown in Fig 1. While retinal layers can clearly be identified in conventional OCT reflectivity images, pigmented tissue (depolarization—yellowish to blueish) can be distinguished from polarization preserving tissue (red) using PS-OCT. As a third channel, OCTA identifies the retinal vascular plexuses. In this paper, the proposed multi-functional approach was used to investigate long-term retinal changes in a very-low-density-lipoprotein-receptor (VLDLR) knockout (Vldlr^tm1Her^, Vldlr^-/-^) mouse [6, 30].

The VLDLR mutant mouse model is a popular strain for studies of retinal neovascularization associated with impaired vision and blindness in ophthalmic diseases such as neovascular AMD and its subforms [6]. It was particularly useful as a model for a subset of neovascular AMD called retinal angiomatous proliferation (RAP), where the angiomatous proliferation has its origin in the retina and not in the choroid [31]. In the past, the VLDLR knockout mouse model was associated with multiple retinal pathogenic features such as neovascular growth, subretinal hemorrhages, retinal-choroidal anastomosis, retinal pigment epithelium (RPE) disruption and hyperplasia, photoreceptor degeneration as well as subretinal fibrosis [6, 30, 32–34]. The majority of these features were identified ex vivo at various ages ranging from postnatal day 7 (P7) [33] to 2 years of age [30]. While the model was studied extensively using histology and immunochemistry, only few in vivo studies were performed and comprised fundus photography, fluorescein angiography and electroretinography [6, 30, 32].

In contrast to previous studies of the VLDLR knockout mouse model, in this work we report the first in vivo long-term follow-up study ranging from P26 to P328. Furthermore, we demonstrate simultaneous volumetric morphological and functional imaging providing intrinsic contrast of spontaneous pathophysiological processes, such as melanin migration during neovascularization, for rodent models. This work shows the potential of multi-functional OCT in preclinical studies of regulative processes in mutant mice which develop pathologies similar to human ophthalmic diseases such as AMD or glaucoma.

In the following section, the multi-functional OCT approach, the animals used, as well as the evaluation process and criteria are defined. Subsequently, the major results of the longitudinal evaluation are highlighted, such as changes in the morphological structure of the retina, melanin migration and retinal neovascularization. The last sections of the paper include a discussion of the paper in context with the inter-disciplinary research fields of biomedical imaging, ophthalmology and basic research as well as a conclusion.

## Materials and methods

### Image acquisition

For the purpose of multi-functional retinal imaging, a custom made PS-OCT system was utilized. A detailed description of the system was previously published by Fialová et al. [28]. In short, the OCT system is based on a polarization-sensitive Michelson interferometer [35, 36], where the sample is illuminated by circularly polarized light and, after interference with linearly polarized light at 45° from the reference arm, the signal is split into two orthogonal polarization states (*S*_*V*_, *S*_*H*_) and detected by two custom-made spectrometers (Basler sprint camera, 3072 *pixels*). The spectral domain (SD) OCT system (*central* *wavelength* λ_*c*_ = 840 *nm*; *bandwidth* Δλ = 100 *nm*) is operating at an A-scan rate of 83 kHz and provides a field-of-view of up to 30° × 30°. The system is tailored for retinal imaging of the posterior rodent eye with an axial resolution of 3.8 *μm* [28].

**Fig 2:**
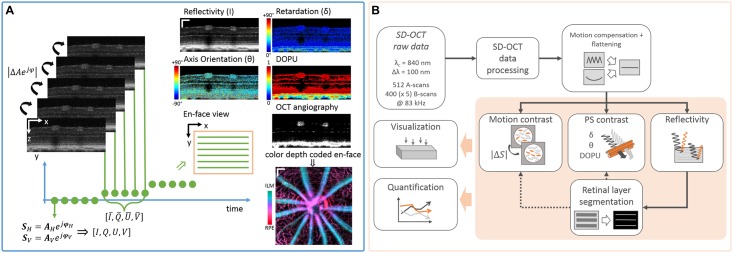
Modified raster scanning and proposed post-processing pipeline for multi-functional OCT imaging. (A) Repeated raster scanning enables the application of motion contrast algorithms for the visualization of blood vessels and for speckle noise reduction in the reflectivity and PS contrast images. Furthermore, the temporal window was used to calculate high-resolution degree-of-polarization-uniformity (DOPU) images. (scale bar: 100 *μm*) (B) The proposed post-processing pipeline can mainly be divided into pre-processing (SD-OCT data processing, motion compensation), multi-functional contrast processing, retinal layer segmentation and the qualitative as well as quantitative evaluation.

High resolution multi-functional image contrast was achieved by a repeated raster scanning protocol. One B-scan, comprising 512 A-scans, was repeated 5 times at each B-scan position. Repeated scanning was performed at 400 B-scan positions covering an area of approximately 1 *mm* × 1 *mm* on the mouse retina. This resulted in a volumetric dataset comprising 1536 × 512 × 400(× 5) pixels *z* × *x* × *y*(× *t*/*repetitions*) after Fourier transform. A sketch of the multi-functional scanning protocol to achieve a threefold contrast is depicted in Fig 2A.

### Multi-functional OCT

In conventional OCT, the contrast is defined by the intensity of the light backscattered at various depths along one A-scan. As outlined in the introduction, various functional extensions were developed in the past 25 years of OCT development. In this work, we defined multi-functional OCT contrast as a threefold contrast comprising reflectivity, PS contrast and motion contrast.

#### Reflectivity

To reduce speckle noise in the reflectivity images *I*, each set of repeated B-scans was averaged after motion compensation [37]. The high resolution reflectivity images (non-averaged) in this work were used for compensating respiration-induced motion artefacts, retinal layer segmentation (averaged images) as well as for the qualitative and quantitative analysis of the experiment.

#### PS contrast

PS-OCT can be used to measure the Stokes vector *S* = (*I*, *Q*, *U*, *V*) for each sample point which describes the polarization state of the backscattered light [38]. Averaging of the Stokes vector along *t* was done to reduce noise in the PS-OCT images as previously presented by Sugita et al. [39]. Hence, phase retardation *δ* and fast axis orientation *θ* were calculated with an increased signal-to-noise ratio (SNR) and this allowed the visualization of birefringent structures. A further PS-OCT parameter is the degree-of-polarization-uniformity (DOPU) [40]. It describes the scrambling of the polarization state, i.e. change of the Stokes vector, in the neighbourhood of a sample point. The parameter values range between 0 (depolarization) and 1 (polarization preserving) which are shown in DOPU images from blue to red, respectively. To achieve high resolution DOPU images, a spatio-temporal window of 3 × 9 × 5 pixels (*z* × *x* × *t*) was used. The DOPU value in this window was calculated only if more than 80% of the pixels in the window exceeded an intensity threshold defined as $\bar{s_{b}}+3\cdot \sigma (s_{b})$, where *s*_*b*_ is determined as the background intensity level in the vitreous. Pixels which did not meet this criterion were treated as NaN (Not a Number) and shown in black. In this work, we focused on the DOPU parameter which enabled the identification of depolarizing tissue such as the RPE, the choroid (CH) or migrated intraretinal melanin pigments. PS parameters such as the retardation *δ* and axis orientation *θ* were not further investigated in this work but could be addressed in future investigations.

#### Motion contrast

Moving red blood cells backscatter the incident light and cause fluctuations in amplitude *A* and phase *φ* when observed at a single location. Hence, the lapse of time in one set of B-scans can be exploited to differentiate between dynamic and static scatterers. This approach, so called OCTA, offers a label-free, 3D method to visualize multiple plexus (network of blood vessels), such as the inner retinal vessels in the NFL or smaller capillaries in the outer plexiform layer (OPL). Different algorithms exist to detect the variations in the backscattered signal *S* = *A*⋅*e*^*jφ*^ [12, 13, 15]. In this work, the motion contrast was calculated by the averaged magnitude of the complex differences between consecutive B-scans after compensating for bulk motion and removal of frames with severe motion. OCTA was used to visualize both retinal and sub-retinal blood flow.

### Image processing and analysis

Longitudinal preclinical studies demand an efficient image processing and analysis pipeline as a vast amount of image raw data has to be processed (≈ 280 datasets ≈ 3.3 *TB* in this study). Hence, a custom-made fully automated image processing pipeline and a semi-automated evaluation framework was developed. An outline of this pipeline is shown in Fig 2B. The proposed pipeline can mainly be split into four parts which include (I) pre-processing, (II) retinal layer segmentation, (III) multi-functional contrast processing and (IV) evaluation.

#### Pre-processing

After conventional SD-OCT raw data processing (mapping from λ to *k* space, dispersion compensation, Fourier transform) of the two orthogonally polarized OCT signals *S*_*V*_ and *S*_*H*_, the reflectivity *I* was calculated as *I* = |*S_V_*|^2^ + |*S_H_*|^2^. The logarithmic scaled and dynamic range adjusted reflectivity images were subsequently used for automated cropping, motion compensation and flattening of the data. Due to the acquisition time of approximately 15 s, respiratory motion of the animal was present as primarily axial shifts of B-scans with an approximate frequency of 1 Hz. To compensate for resulting artefacts (wavy appearance of the retina, artificial horizontal lines in en-face views) the maximum gradient of the cumulative sums along each A-line was determined to approximate the shape of the retina. A polynomial surface was fitted to this approximation to refine the retina estimate, thereby taking outliers into account. Axial shifts were subsequently calculated by comparing the retinal shape approximation in consecutive B-scans, and the retina was flattened by a transformation of the polynomial surface to a plane. Subsequently, the motion compensated and flattened reflectivity images were averaged for each set of repeated B-scans to reduce speckle noise. Hence, the resulting reflectivity $\bar{I}$ dataset consisted of *n*_*z*_ × 512 × 400 pixels (*z* × *x* × *y*), where *n*_*z*_ is the number of pixels along the A-line and determined by the cropping procedure.

#### Retinal layer segmentation

**Fig 3:**
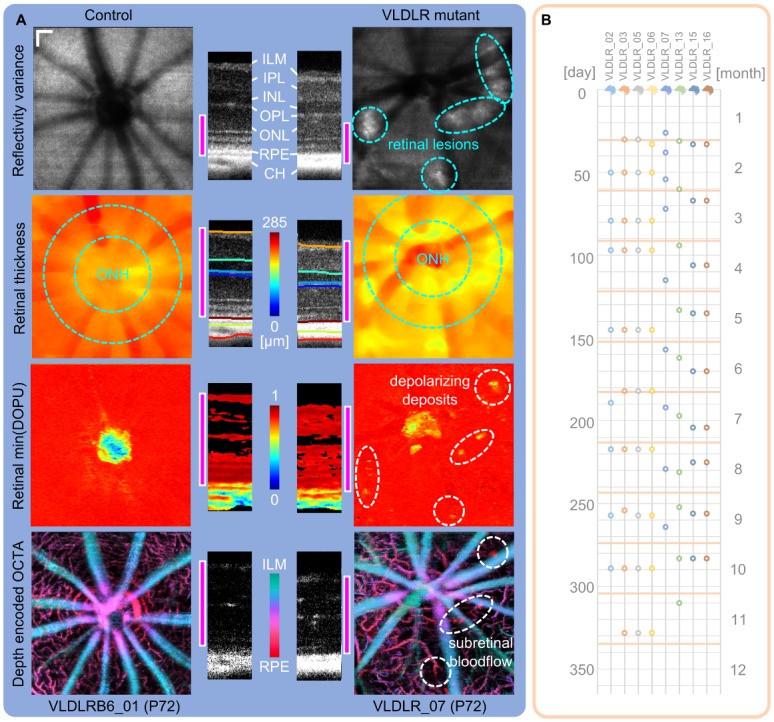
Multi-functional OCT images and timeline of measurements. (A) Contrast specific projections were used for a quick identification of retinal abnormalities in the longitudinal investigation of the VLDLR mutant retina. An age-matched (P72) control mouse is compared to a Vldlr^-/-^ mouse. Retinal lesions can be identified in reflectivity variance projections of the outer retina. Retinal thickness changes are present, especially at the location of lesion sites and is evaluated in a circumpapillary region excluding the optic nerve head (ONH). Depolarizing deposits within the retina of the Vldlr^-/-^ mouse can be identified by a minimum DOPU projection. OCTA provides 3D contrast of the retinal microvasculature. Three plexuses can be visualized in the animals and pathological intraretinal neovascularization in the ONL was observed. (scale bar: 100 *μm*; pink bar: depth range which was used for the respective parameter’s en-face projection) (B) Image acquisition timeline representing the measured mutant mice at the respective postnatal days by a circle. For the evaluation process, the individual measurements were clustered per month.

For the visualization of different regions within the retina (sub-volumes, slabs) an automated retinal layer segmentation algorithm was used to determine interfaces between retinal layers. In this work, a graph-based approach for two dimensional (2D) images, which was previously presented by Srinivasan et al. [41], was adapted to our needs. In short, each pixel in a B-scan was represented as a node and the edges of the graph connecting neighbouring pixels were determined by a cost function primarily based on the axial image gradient. A layer was segmented by minimizing the cost function while travelling across the B-scan, iteratively for each layer. In addition to the initial 2D segmentation, we implemented a 3D refinement step to correct for segmentation inaccuracies due to image quality variations or presence of pathological structures. The algorithm was adapted to segment the following transitions and layers (anterior to posterior—Fig 3A):

1. Inner limiting membrane (ILM),
2. Inner plexiform layer (IPL) → inner nuclear layer (INL),
3. INL → OPL,
4. OPL → outer nuclear layer (ONL),
5. Anterior RPE and
6. Posterior CH boundary.

An additional layer was determined by a surface fit between the RPE and the CH as a reference plane in case of lesions or large choroidal vessels. Exemplary reflectivity B-scans and their corresponding segmentation are shown for a 72 days old Vldlr^-/-^ mouse and for an age-matched control mouse in Fig 3A.

#### Visualization of multi-functional OCT image contrast

Contrast specific projections were determined in order to detect pathological features in the rodent’s posterior eye and to allow a quick interpretation of longitudinal image data. A comparison and overview of the different projection types are shown for a VLDLR mutant mouse and an age-matched control mouse at P72 in Fig 3A.

#### Outer retina reflectivity variance projection

Lesion development was observed in Vldlr^-/-^ mice. To identify the lesion sites and enable tracking over time, a slab of 50 *pixels* (corresponding to ≈100 *μm*) was defined above the RPE-CH complex fit. While the variance of the reflectivity signal was expected to be constant in non-lesion sites, pathological destruction of photoreceptors and formation of highly reflecting lesion sites were believed to lead to a higher variation in the reflectivity signal. Hence, the intensity variance along *z* was calculated within the 50 *pixels* slab to reveal the lesions in an en-face projection.

#### Retinal thickness map

Retinal thickness maps were calculated as the axial distance between ILM and the RPE upper boundary.

#### Retinal minimum DOPU projection

Apart from the RPE and remainders of melanin pigments around the optic nerve head (ONH), which manifest during development of the hyaloid canal, the rodent retina can be characterized as non-depolarizing tissue [29]. To detect pathological pigmentation within the retina, a minimum DOPU projection (pigmented tissue corresponds to low DOPU values) was used to identify changes in the polarization characteristic of the retina.

#### Depth-color coded OCTA

To detect pathological intraretinal blood flow and morphological changes in the vascular plexuses, depth color-coded OCTA en-face projections were determined. Here, blood flow is visualized on a color scale from greenish to red dependent on the axial location between the ILM and the RPE.

#### Evaluation

After automated post-processing which included all previously described steps, a semi-automated evaluation of the multi-functional image data was performed. The intermediate results such as the retinal layer segmentation were visually inspected for errors. In case of a low-quality acquisition, e.g. due to severe motion of the animal, or in case of segmentation errors, the measurement was rejected and was not included in the evaluation process. Subsequently, for each measurement, the following seed points were set manually:

- ONH position: The center of the ONH was identified in a maximum intensity projection image of the RPE-CH complex slab.
- Retinal lesions: Each lesion was annotated by choosing the most anterior pixel of a lesion in the reflectivity images.
- Depolarizing deposits: Depolarizing regions were selected by choosing the most anterior deposit in the depolarizing region-of-interest (ROI).

The retinal thickness was evaluated in a circumpapillary annulus with an outer radius of 225 *pixels* ≈ 450 *μm* centered at the ONH, excluding a circular region with a radius of 125 *pixels* ≈ 250 *μm*. The area of evaluation is indicated by cyan colored circles in Fig 3A. The mean retinal thickness was determined only if at least 75% of the pixels in the annulus were covered by the acquisition.

### Animals

A homozygous breeding pair of mice for the Vldlr^tm1Her^ mutation was purchased from The Jackson Laboratory (Stock No: 002529). Animals were bred and kept under controlled lighting conditions (12 hours light, 12 hours dark) at the Division of Biomedical Research at the Medical University of Vienna. Both eyes of 8 female Vldlr^-/-^ mice were studied within the age range of P26 to P328. A detailed acquisition timeline is depicted in Fig 3B. The mice were imaged approximately every 4 to 6 weeks. C57BL/6 mice were crossbred with homozygous VLDLR mutant mice to establish a control group. Four female control mice were imaged at P44, P72 and P213. All animals were anaesthetized during the experiment using an isolflurane/oxygen mixture for 15 to 30 minutes. Tropicamide and phenylephrine were used for pupil dilation, and the cornea was kept moisturized using artificial tear eye drops. The animals were sacrificed using a sodium pentobarbital injection (intraperitoneal) at the endpoint of the experiment. All experiments were performed in accordance with the Association for Research in Vision and Ophthalmology Statement for the Use of Animals in Ophthalmic and Vision Research and under a protocol approved by the ethics committee of the Medical University of Vienna and the Austrian Federal Ministry of Science, Research and Economy (GZ:BMWFW-66.009/0121-WF/V/3b/2016).

### Histology

Retinal changes tracked during aging of the Vldlr^-/-^ mice were correlated to histology. Three 11-month-old mice (*VLDLR*_03, *VLDLR*_05, *VLDLR*_06) were sacrificed after imaging, and both eyes were enucleated and processed for histological analysis. Frozen and paraffin sections were obtained in precise alignment with OCT images using major blood vessels around the ONH as orientation markers. The eyes were counterstained with DAPI (4’,6-diamidino-2-phenylindole) and examined using epifluorescence and differential interference contrast (DIC) microscopy.

### Statistical analysis

To investigate the longitudinal effect of time on the retinal thickness and the number of retinal lesions, a repeated measures analysis of variance (ANOVA) with post hoc tests was performed. Bonferroni correction was used in the post hoc tests to adjust the level of significance for multiple comparisons. A comparison between the VLDLR mutant mice and the control group was performed using the Mann-Whitney U test. P-values < 0.05 were considered significant, with *p < 0.05, **p < 0.01 and ***p < 0.001. In random cases of mice not cooperating missing values occurred in the longitudinal dataset. To overcome incomplete data in the statistical analysis, elimination of variables and observations as well as imputation was used. Variables (time/month) lacking more than 25% of the observations (mouse eye) were eliminated. Hence, the observations at the months 1, 7 and 11 were eliminated and month 2 was set as the baseline for the longitudinal statistical analysis. Furthermore, VLDLR_007 (left eye) was eliminated from the set of observations as more than 25% of the variables were missing. To impute missing values (10 for lesion analysis; 12 for thickness analysis; 120 total values), a linear local trend was assumed. Each eye of one mouse was assumed as an individual observation and latest measurements were chosen during the monthly clustering in case of more than one measurement per month. All statistical analyses were performed using IBM SPSS Statistics Version 24.

## Results

### Retinal lesions sites

**Fig 4:**
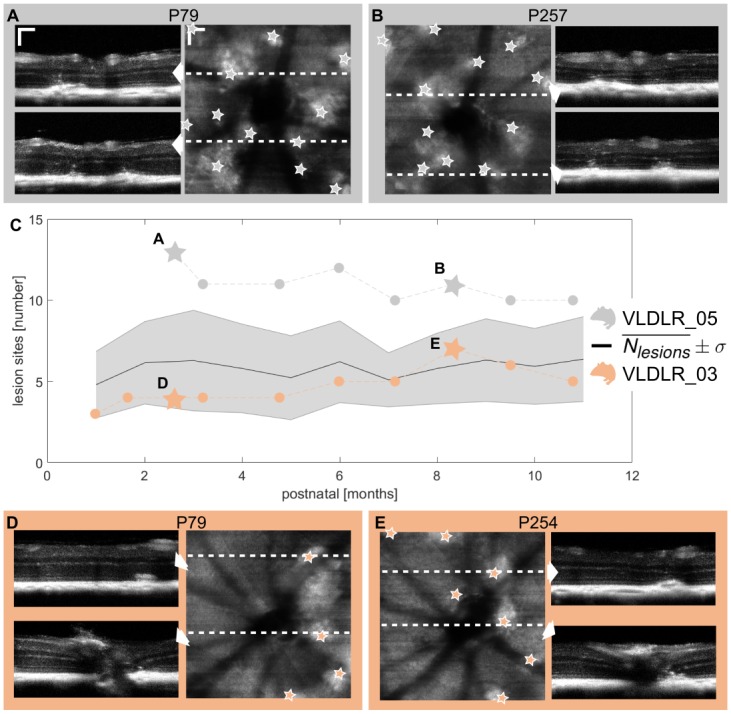
Long-term follow up of retinal lesion development. (A,B) Retinal lesions identified in reflectivity B-scans and en-face reflectivity variance projections at two different dates of measurement for VLDLR_05. (C) Mean number of retinal lesions and standard deviation clustered for each month during the longitudinal study. Retinal lesions were present in all animals at the initial measurement. Once the lesions were manifested, the number of lesions did not change significantly. The changes in the number of lesions are due to variations in the measurement position. The exact numbers of lesions are shown for two exemplary mice in gray and orange which correspond to the images shown in (A,B) and (D,E) respectively. The grey and orange stars mark the date of the measurement for the exemplary shown images. (D,E) Exemplary lesions identified for VLDLR_03. (scale bar: 100 *μm*.)

Retinal lesions develop in the VLDLR knockout mouse model between P14 and P21 [6, 30, 34]. In the reflectivity B-scans, lesions can clearly be identified as bumpy alterations of the hyperreflective RPE-CH complex, see Fig 4A. A minimum of 1 lesion (*mean* ± *σ*; 5 ± 2) in the field-of-view of 1 *mm*^2^ around the ONH was present in all animals at the initial measurement at P36 (±13). The average number of lesions ($\bar{N_{lesions}}\pm \sigma$), clustered for every month, is shown in Fig 4C. Retinal lesions identified in the en-face projections as well as exemplary B-scans are shown for two mice at two different dates of measurement in Fig 4A, 4B, 4D and 4E. The peaks of the lesions are indicated by stars. The variations in numbers of lesions were mainly due to variations of the imaging position. The number of eyes *N*_*eyes*_ evaluated per month, the average number of lesions per eye $\bar{N_{lesions}}$ and the corresponding standard deviation *σ* (*N*_*lesions*_) are listed in Table 1. The statistical analysis showed that there was no significant change in the number of lesions detected during the longitudinal evaluation between the ages of 2 months and older (p = 0.131). We did not encounter lesion development in the control group, i.e. no lesions were detected at any of the investigated ages (2, 3 and 8 months). Furthermore, we investigated the distribution of the lesions in the retina. The average Euclidean pairwise distance for each measurement between the individual lesions remained constant over time. The average nearest neighbour of an individual lesion was determined as 335 (±123) *μm*.

**Table 1:** Number of retinal lesions and depolarizing deposits.

Month	1	2	3	4	5	6	7	8	9	10	11
*N*_*eyes*_	5	14	14	15	13	14	10	15	16	14	8
$\bar{N_{lesions}}$	4.80	6.21	6.29	5.80	5.23	6.21	5.10	5.80	6.31	5.93	6.38
*σ* (*N*_*lesions*_)	2.05	2.52	3.10	2.73	2.59	2.52	1.66	2.18	2.55	2.34	2.62
*N*_*depos*_	0	26	53	63	32	55	38	72	69	45	33
$\bar{N_{depos}}$	0	1.86	3.79	4.20	2.46	3.92	3.80	4.80	4.31	3.21	4.13
*σ* (*N*_*depos*_)	0	1.35	2.36	2.68	1.39	1.98	2.35	2.81	2.30	2.04	2.03

*N*_*eyes*_, number of eyes; $\bar{N_{lesions}}$, average number of lesions per eye; *σ* (*N*_*lesions*_), standard deviation of lesions per eye; *N*_*depos*_, total numbers of depolarizing retinal deposits; $\bar{N_{depos}}$ average number of depolarizing deposits per eye; *σ*(*N*_*depos*_) standard deviation of depolarizing deposits per eye.

### Longitudinal changes of retinal thickness

**Fig 5:**
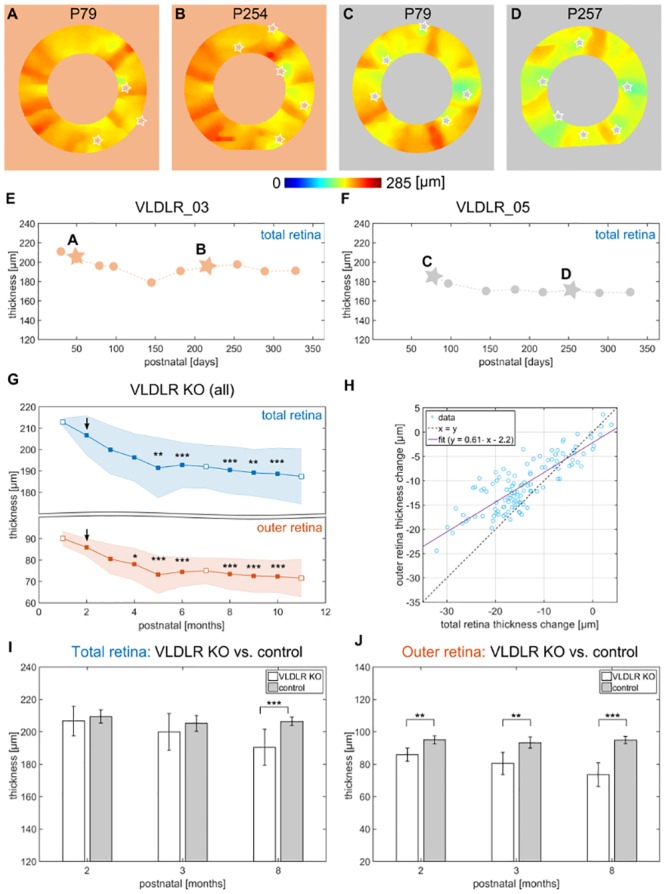
Longitudinal changes of retinal thickness. (A-D) Exemplary total retina (TR) thickness maps extracted in an annulus of 200 *μm* width for VLDLR_03 (A,B) and VLDLR_05 (C,D). Variations can be identified around the major retinal blood vessels as well as around lesion areas (lesions are depicted with stars). (E,F) Longitudinal evaluation of the TR thickness for the exemplary mice shown in (A-D). (G) The mean TR thickness clustered by month showed a significant thinning between the age of 2 and 5 months and older, while a significant thinning of the outer retina (OR) was observed between the at age of 2 and 4 months. The data at the age of 2, 7 and 11 months (white markers) were excluded from the statistical analysis and the age 2 months was used as a baseline. (H) The correlation analysis between the decrease in thickness of the OR versus the TR showed a strong correlation, *ρ* = 0.84. (I) Comparison between VLDLR mutant mice and control group at the ages 2, 3 and 8 months. A significant difference was observed in the TR thickness at the age of 8 months, where the mutants’ retinas were thinner, while the retinas of the control mice remained rather constant. The OR thickness was significantly reduced at the age of 2 months between the VLDLR knockout (KO) mice and the controls. (*p < 0.05; **p < 0.01; ***p < 0.001.)

The retinal thickness *t*_*R*_ was evaluated in an annulus around the ONH as indicated in Fig 3A. Total retinal (TR) thickness maps in the annulus for two exemplary mice at two different dates are shown in Fig 5A, 5B, 5C and 5D respectively. A variation in the thickness maps can be identified around the major retinal blood vessels (increase of *t*_*R*_) and around retinal lesions (decrease of *t*_*R*_; lesions inside the annulus are indicated by stars). The retinal thickness was defined as the average thickness value in the annulus. The longitudinal data (TR thickness) of two exemplary mouse eyes are shown in Fig 5E and 5F. Overall, a decreasing trend of retinal thickness can be observed in the graph of all animals clustered into months in Fig 5G. The statistical analysis confirmed a significant change in the TR thickness (*p* < 0.001) in the longitudinal dataset, whereas a significant (*p* < 0.01) decrease in retinal thickness at the age of 5 months (compared to age 2 months) and older was observed. To investigate the origin of the TR thickness change, the retina was further sub-divided into inner retina (IR), i.e. from the ILM to the OPL, and outer retina (OR), i.e. from the OPL to the RPE. A significant thinning of the OR was observed (*p* < 0.001) and was initially detected between the age of 2 and 4 months, see Fig 5G. A correlation analysis between the decrease (absolute difference to initial measurement) of TR, IR and OR revealed a strong correlation between TR and OR (Pearson correlation coefficient *ρ* = 0.84; *p* < 0.001) and the TR and IR (*ρ* = 0.71; *p* < 0.001) as well as a weak correlation between the OR and the IR (*ρ* = 0.21; *p* < 0.05). The correlation between the TR and OR decrease is shown in Fig 5H. Furthermore, the TR and OR thickness of the mutant mice were compared to the respective thicknesses of the control group at the age of 2, 3 and 8 months. While there was no significant difference in TR thickness detected between the two groups at the age of 2 and 3 months, the total retina was significantly (*p* < 0.001) thinner in the mutant mice at an age of 8 months. The OR thickness was significantly reduced already at the age of 2 months (*p* < 0.01) and even more so at the age of 8 months (*p* < 0.001). There was no trend of a decreasing in TR or OR thickness observed within the first 8 months in the control group, see Fig 5I and 5J. The mean retinal thicknesses of all evaluated eyes clustered for each month are listed in Table 2.

**Table 2:** Long-term changes in retinal thickness.

Group	Month	1	2	3	4	5	6	7	8	9	10	11
**VLDLR mutant**	*N*_*eyes*_	3	13	13	14	13	14	9	15	16	14	8
	$\bar{t_{TR}}\;[\mu m]$	212.80	206.62	199.84	196.30	191.46	192.72	191.99	190.35	189.13	188.62	187.41
	σ (*t_TR_*) [*μm*]	1.74	9.14	11.36	11.13	14.12	10.60	10.16	11.08	10.92	12.03	12.94
	$\bar{t_{OR}}\;[\mu m]$	90.11	85.94	80.49	78.03	73.17	74.47	75.00	73.50	72.58	72.27	71.55
	σ (*t_OR_*) [*μm*]	3.38	4.00	6.94	7.44	8.72	6.77	6.01	7.30	7.62	7.43	8.84
**Control**	*N*_*eyes*_		6	7					8			
	$\bar{t_{TR}}\;[\mu m]$		209.40	205.21					206.43			
	σ (*t_TR_*) [*μm*]		4.10	4.88					2.59			
	$\bar{t_{OR}}\;[\mu m]$		95.05	93.37					94.96			
	σ (*t_OR_*) [*μm*]		2.44	3.54					2.22			

*N*_*eyes*_, number of eyes; $\bar{t_{R}}\;[\mu m]$, average retinal thickness; σ (*t_R_*) [*μm*], standard deviation retinal thickness; TR, total retina; OR, outer retina.

### Migration of depolarizing deposits in the outer retina

**Fig 6:**
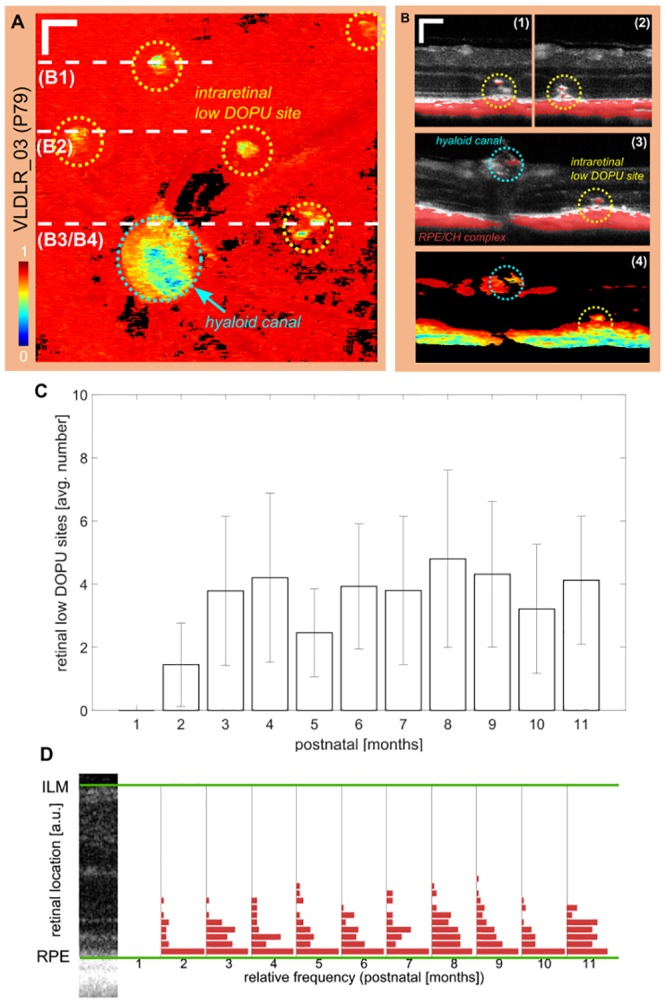
Intraretinal deposits of migrated melanin pigments. (A) Intraretinal minimum DOPU projection of an exemplary mouse at P79. 6 clusters of low DOPU values can be identified. The biggest accumulation (cyan circle) is associated to melanin pigments at the hyaloid canal. The remaining sites are related to melanin migration during lesion development. (scale bar: 100 *μm*) (B) Exemplary reflectivity B-scans with a superimposed thresholded DOPU image (*DOPU* ≤ 0.5) at the lesion sites as shown in (A). Intraretinal deposits with low DOPU can clearly be identified along with disruption of the RPE in the DOPU B-scan of B3. (C) Mean number of low DOPU sites for all eyes clustered monthly. Error bars indicate the standard deviation. (D) The retinal location of the deposits was evaluated and binned into 20 equally spaced slabs between the RPE and the ILM. Most deposits are located close to the RPE and range up to the OPL.

During the development and manifestation of retinal lesion sites evoked by retinal-choroidal neovascularization, melanin accumulations migrate from the RPE-CH complex into the outer retina. Melanin pigments cause a depolarization of light and can be detected by the DOPU parameter [29]. The number of intraretinal depolarizing deposits was evaluated for the long-term follow up experiment. An exemplary intraretinal minimum DOPU projection is shown in Fig 6A for a 79 days old Vldlr^-/-^ mouse. Depolarization around the hyaloid canal can be observed along with 5 other depolarizing sites across the imaged part of the retina. Exemplary reflectivity B-scans overlaid with the corresponding, thresholded DOPU images are shown in Fig 6B1 to 6B3. An exemplary DOPU image, corresponding to the superimposed image in Fig 6B3, is shown in Fig 6B4. Depolarizing deposits in the neighborhood of retinal lesion sites can be observed. The number of regions of depolarizing deposits was evaluated for each measurement. Fig 6(C) shows a graph including the number of depolarizing sites clustered for each month during the longitudinal study. The number of depolarizing deposits peaked after 3 months. In Fig 6D the locations of the depolarizing deposits which were found in the mouse retina are shown on a graph. Each retina was binned into 20 equally spaced slabs between the ILM and the RPE, and the number of depolarizing deposits was counted for each slab. The relative histograms are shown for each month. The deposits were mainly found in the region between the RPE and the OPL. Furthermore, most of them were found close to the RPE which can be associated with the disruption of the RPE during lesion development. The absolute number of lesions *N*_*depos*_ and their average per eye $\bar{N_{depos}}\pm \sigma \;(N_{depos})$ determined along the long-term investigation of the VLDLR mutant mice are shown in Table 1.

### Retinal-choroidal anastomosis

**Fig 7:**
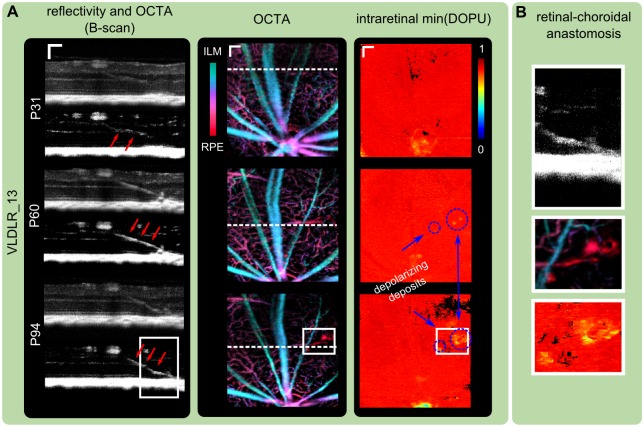
Retinal-choroidal anastomosis. (A) Exemplary reflectivity B-scans and OCTA B-scans (maximum intensity projection of 10 OCTA B-scans) for one VLDLR knockout mouse at three different dates show a bridge-like structure between the OPL and RPE. The lesion was correlated with the presence of blood flow in the corresponding OCTA images (indicated by red arrows in the OCTA images). Furthermore, depolarizing deposits were identified close to the lesion site. (scale bar: 100 *μm*) (B) Enlarged regions as indicated by white rectangles in (A). Color depth encoded OCTA image clearly shows the neovascularization in the outer retina region.

Retinal and choroidal neovascularization (RNV, CNV) develop in the VLDLR mutant mouse model and lead to retinal-choroidal anastomosis [6, 30, 34]. We were able to identify retinal-choroidal anastomosis in all investigated animals in vivo using OCTA. Fig 7(A) shows an exemplary mouse at three ages between P31 and P94. A bridge-like hyper-reflective structure can be identified in the reflectivity images and is even more pronounced in the corresponding OCTA B-scans. The neighboring region in the OCTA en-face images clearly shows the presence of retinal blood flow in the location of the photoreceptors (red in the depth color coded OCTA) at P60 and was even more pronounced in the follow up measurement at P94. Furthermore, this lesion site with presence of blood flow was also correlated to a region with depolarizing deposits. Fig 7(B) shows an enlarged version of the regions denoted with a white rectangle in the corresponding reflectivity, OCTA and DOPU images.

### Correlation with histology

**Fig 8:**
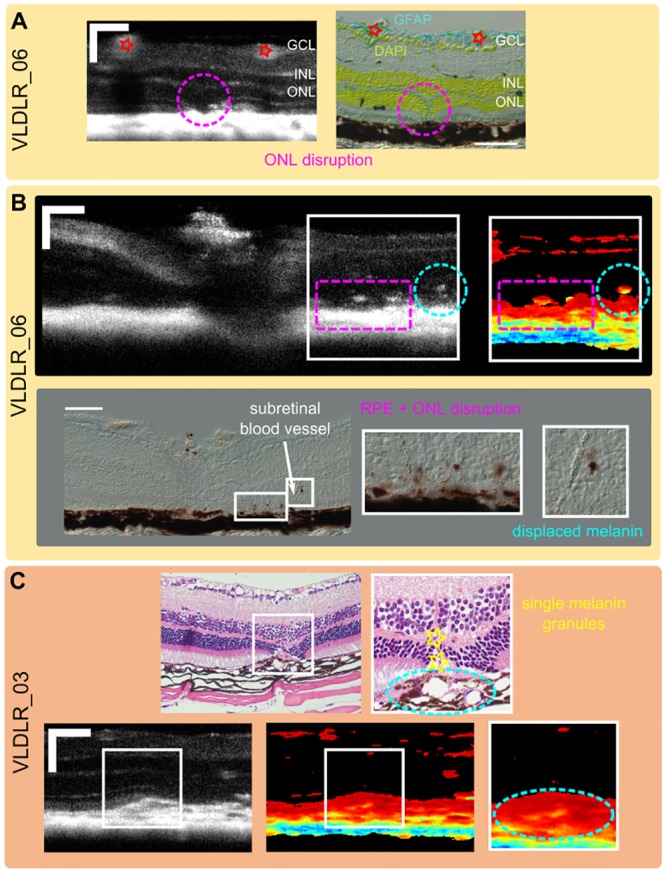
Correlation of multi-functional in vivo OCT measurements with histology. (A) Disruption of the ONL is clearly visible in the reflectivity B-scans and was confirmed by histological sectioning. (B) In vivo PS-OCT measurement was matched with DIC images of a frozen section. Displaced melanin colocalizes with DOPU images showing higher intraretinal depolarization. (C) Single melanin granules could not be visualized by PS-OCT while sites of dense pigments around subretinal fibrous tissue could be matched to regions of low DOPU values. (scale bar: 100 *μm*.)

To correlate the findings of our longitudinal in vivo investigation, histological sectioning was performed for three mice (VLDLR_03, VLDLR_05, VLDLR_06) at the endpoint of the in vivo experiment (11 months). A reflectivity B-scan and a corresponding histological section of a lesion site in the VLDLR knockout mouse are shown in Fig 8A. The reflectivity OCT scan clearly resolves the disruption of the ONL which is confirmed by the histological section. Reflectivity B-scans and corresponding DOPU B-scans through the papilla were matched with DIC images of a frozen section as exemplary shown in Fig 8B. A section which precisely matches the in vivo scan shows that displaced melanin colocalizes with a site of increased depolarization (yellowish) in the severely disrupted ONL. In Fig 8C, an HE-stained paraffin section through a lesion site is showing a disrupted ONL and outer limiting membrane (OLM) as well as fibrovascular proliferations surrounded by pigments. While the disruption could be nicely correlated to the OCT reflectivity scan, the single melanin granules in the vicinity of newly formed blood vessels in the ONL are clearly not resolved by in vivo PS-OCT. Nevertheless, sites of dense pigment surrounding subretinal fibrous tissue (oval) nicely correlate with lower values in the DOPU images.

## Discussion

In this work, we demonstrated a multi-functional OCT approach to investigate longitudinal changes in a VLDLR mutant mouse model which are mainly caused by retinal-choroidal neovascularization. RNVs, which develop between P14 and P21 [6, 30, 34], were identified as hyper-reflective sites in OCT reflectivity images. The retinal lesions were detected for all eyes beginning with the initial measurement around P36 (±13) and did not change significantly until the endpoint of the experiment which was between 10 to 11 months of age. The functional longitudinal investigation of retinal lesions showed the presence of abnormal blood flow and increased depolarization caused by migrated melanin pigments. Intraretinal depolarization was mainly present in two forms. On the one hand, large areas of increased depolarization were observed close to the RPE, and on the other hand, isolated depolarizing deposits were identified in the ONL. Both forms were confirmed by precisely matching histological sections of 3 animals to the respective in vivo OCT scans. Depolarizing spots were correlated with clusters of melanin pigments migrating into the retina during the development of retinal-choroidal neovascularization. This migration of RPE cells was previously described by Heckenlively et al. [6] in histological sections at 9 months of age, by Hu et al. [30] at 10 months of age and in the work of Li et al. [34] between 6 to 8 weeks of age. An increased number of depolarizing clusters close to retinal lesions started to be detectable by PS-OCT at an age of 3 months. In general, it seemed that the lesion formation reaches a peak at an age of 5 months, and it remains rather constant for the subsequent 6 months of investigation in the OCT images. This reflects in the presence of increased depolarizing deposits, the significant decrease of retinal thickness until the age of 5 months and the clear presence of retinal-choroidal anastomosis at this age. A decreased thickness of the ONL was previously described by Chen et al. [32] at the age of 3 months ex vivo. Hu et al. [30] also identified the disruption and thinning of the ONL as well as the complete extinction around lesion sites at 10 months of age. Measurement of the decrease in retinal thickness for both the total retina and the outer retina revealed a strong correlation (*ρ* = 0.84), which suggests that the decreased retinal thickness, measured using multi-functional OCT, was mainly caused by the destruction of the outer retina (see Fig 5). The evaluation of the total and outer retinal thickness of the control group showed no decrease until the age of 8 months. This analysis is supported by experiments evaluated in literature, where a retinal thinning in wildtype mice is observed only with an age of 10 months and older [42]. Furthermore, the comparison with the control group revealed that the outer retina was significantly reduced with an age of 2 months, while the total retina was not.

In vivo analyses regarding the visualization of blood flow in the VLDLR knockout mouse model were addressed by Heckenlively et al. [6] and Chen et al. [32] using fluorescein angiography at 3, 6 and 8 weeks as well as at the age of 6 months. Heckenlively et al. [6] noticed an increase of neovascular dye leakage until the age of 6 months. The presence of lesions was detected in our study with the initial measurement in the conventional OCT reflectivity images. The presence of abnormal vasculature was evident in the OCTA images around P60 and was also confirmed at later stages (see Fig 7). The process of retinal-choroidal anastomosis was clearly identified in all animals using OCTA in vivo non-invasively. This suggests that the functional extension of OCTA can be beneficial to detect and study neovascularization in preclinical research in 3D. Recently, this was first demonstrated by Liu et al. [43] in a mouse model with laser-induced neovascularization. To the best of our knowledge, this work is the first work to demonstrate OCTA for the visualization of spontaneous neovascularization in animal models.

Our experimental evaluation showed that the expansion of conventional OCT to multi-functional OCT is beneficial for advanced preclinical retinal imaging with additional intrinsic contrast. The threefold contrast enables a multi-parametric analysis of pathological processes such as retinal-choroidal anastomosis, where it was possible to image melanin migration (with PS-OCT) during the development of new blood vessels (using OCTA). A quantitative interpretation of the DOPU values, which was left out in this study, but was investigated in detail in our previous work [29] for rats, shows the potential of PS-OCT for basic research questions regarding the RPE and its development during the process of aging. Neovascular symptoms causing vision loss are in focus of clinical studies using OCTA [44, 45]. OCTA enables the study of the morphological changes in vascular plexuses of rodents on a capillary scale. This work suggests that OCTA in preclinical research enables the assessment of neovascular development in genetic rodent models in order to better understand genetically regulated processes. OCTA offers in vivo visualization of the vasculature in a 3D fashion which is not possible in the current gold standard of fluorescein angiography. The determination of absolute blood flow and blood oxygen saturation could further broaden the range of biomarkers in preclinical studies. Recent developments of OCT technologies for small animal imaging have shown promising results in the field of Doppler OCT [25] as well as using visible-light OCT [26].

This work demonstrates that multi-functional OCT might be a powerful tool for future basic research in ophthalmology and may also have a high impact in other disciplines. For example, Leahy et al. [46] demonstrated the visualization of cortical blood flow in rodents, which shows the potential of OCT in the field of neuro-imaging. The non-invasive characteristic not only allows a direct comparison of individual mice at various ages, it also significantly reduces the numbers of animals used in preclinical research and therefore OCT supports the principles of 3 Rs in basic research which are to replace, to reduce and to refine the use of animals [47]. In the present work, 8 mutant mice were imaged in total at 75 different time points. Hence, with the current gold standard of histological sectioning almost the 10-fold number of animals would have been sacrificed. Of course, resolution is sacrificed too when comparing OCT to histology, however our correlation with histology suggests that OCT and histology can be used complementary to each other and foster the identification and understanding of a multi-functional set of parameters enabling the discovery of early biomarkers.

The VLDLR knockout mouse model provides a model which develops phenotypic traits, associated to symptoms in wet AMD [6], RAP [33, 34] and proliferative macular telangiectasia [48]. Suppression of neovascularization in the VLDLR mutant mouse model was recently investigated by different groups. Hua et al. [48] administrated Resveratrol orally before and after disease onset and were able to reduce vascular lesions by suppression of vascular endothelial growth factor (VEGF) transcription. In order to address the aberrant increase of retinal VEGF in the VLDLR mutant mice, Dorell [49] et al. and Zhou et al. [50] injected nanoceria intravitreally which led to an inhibition of pathological vascular lesions. Westenskow et al. [51] compared *α*-miR-132 to VGEF-trap, comparable with a commercially available medication, to investigate the role of the vascular endothelial Ras pathway in neovascular sprouting. Intravitreal injections lead to a significant decrease in both lesion area and number of tufts. A topical anti-angiogenic agent (HL-217) was developed by Kim et al. [52]. In their work, the platelet derived growth factor (PDGF) was addressed and the effect of topical application of HL-217 was investigated by measuring the neovascular area. HL-217 showed to be an effective inhibitor of subretinal neovascularization as neovascular area in the retina was significantly reduced. These recent developments targeting the VLDLR mutant mouse model reveal the importance of future preclinical research and the development of appropriate animal models. Investigation of basic regulative processes by an extensive study of appropriate animal models of ophthalmic diseases can foster the development of molecular approaches for the treatment of retinal angiogenesis in ophthalmic diseases. OCT and its functional extensions might be a powerful diagnostic tool for interventional studies where the success of a treatment should be studied in vivo in a longitudinal fashion.

## Conclusion

In this work, we presented a multi-functional OCT approach for investigating retinal changes in a VLDLR knockout mouse model. The multi-functional image contrast comprises reflectivity, PS contrast and motion contrast based OCTA. VLDLR mutant mice were imaged in a follow-up study (from 4 weeks to 11 months) every 4 to 6 weeks. The longitudinal evaluation revealed the development of neovascular lesions, peaking in the period between 3 to 5 months, which lead to a significantly decreased retina, manifestation of intraretinal melanin pigments and presence of retinal-choroidal anastomosis. Histological sections were correlated to in vivo OCT images in order to identify and confirm retinal changes during the long-term follow up study. Multi-functional OCT, as demonstrated in this work, showed its potential to identify various aspects during spontaneous neovascularization with a threefold intrinsic contrast and suggests that OCT and its functional extensions can be integrated to establish a promising tool for basic research in ophthalmology and related disciplines.
